# Galectin-1 is required for the regulatory function of B cells

**DOI:** 10.1038/s41598-018-19965-z

**Published:** 2018-02-09

**Authors:** R. Alhabbab, P. Blair, L. A. Smyth, K. Ratnasothy, Q. Peng, A. Moreau, R. Lechler, R. Elgueta, G. Lombardi

**Affiliations:** 1 Present Address: Infectious Disease Unit & Division of Applied Medical Sciences, King Fahad Centre for medical research, King Abdulaziz University, Jeddah, Saudi Arabia; 2 Division of Transplantation Immunology & Mucosal Biology, King’s College London, King’s Health Partners, Guy’s Hospital, London, SE1 9RT UK; 3 Present Address: Centre for Rheumatology, Division of Medicine, University College London, London, WC1E 6JF UK; 4 Present Address: School of Health, Sports and Biosciences, University of East London, Stratford, E15 4LZ UK; 5 Present Address: Centre de Recherche en Transplantation et Immunologie UMR 1064, INSERM, Université de Nantes, CHU, Nantes, France

## Abstract

Galectin-1 (Gal-1) is required for the development of B cells in the bone marrow (BM), however very little is known about the contribution of Gal-1 to the development of B cell regulatory function. Here, we report an important role for Gal-1 in the induction of B cells regulatory function. Mice deficient of Gal-1 (Gal-1^−/−^) showed significant loss of Transitional-2 (T2) B cells, previously reported to include IL-10^+^ regulatory B cells. Gal-1^−/−^ B cells stimulated *in vitro* via CD40 molecules have impaired IL-10 and Tim-1 expression, the latter reported to be required for IL-10 production in regulatory B cells, and increased TNF-α expression compared to wild type (WT) B cells. Unlike their WT counterparts, T2 and T1 Gal-1^−/−^ B cells did not suppress TNF-α expression by CD4^+^ T cells activated *in vitro* with allogenic DCs (allo-DCs), nor were they suppressive *in vivo*, being unable to delay MHC-class I mismatched skin allograft rejection following adoptive transfer. Moreover, T cells stimulated with allo-DCs show an increase in their survival when co-cultured with Gal-1^−/−^ T2 and MZ B cells compared to WT T2 and MZ B cells. Collectively, these data suggest that Gal-1 contributes to the induction of B cells regulatory function.

## Introduction

B cells are generally thought to contribute to graft rejection through the production of alloantibody and due to their capacity to efficiently present alloantigens^1^. However, clinical trials in which B cell depletion was used as part of the therapy, have suggested that B cells can contribute to the regulation of the recipient immune responses leading to allografts tolerance^2–4^. Several groups have reported that certain subsets of B cells reduce inflammatory responses in mice, and that autoimmune diseases and transplant rejection can be exacerbated in their absence^5–11^. Various B cell subsets with regulatory functions have been identified such as T2 B cells^12^, CD5^+^ CD1d^+^ B cells^13,14^, Tim-1^+^ B cells^11^, or MZ B cells^15,16^. IL-10 production has been recognized as one of the key mediators of the regulatory functions of these B cell subsets. We recently reported that B cells were capable of prolonging allogenic skin graft survival^10^. Adoptive transfer of T2 and T1 B cells, but not follicular (FO) B cells, from naïve mice housed in conventional animal facilities prolonged the skin allograft survival^10^. B cell capacity to delay skin graft rejection was lost when B cells were derived from IL-10^−/−^ mice^10^. Furthermore, the transfer of T2 B cells from mice rendered tolerant to MHC-class I mismatched skin grafts by donor splenocyte transfusion and anti-CD40L treatment, but not naïve nor alloantigen experienced T2 B cells, can prolong skin graft survival and suppress T cell activation^17^. Ding *et al*. identified T-cell immunoglobulin and mucin domain 1 (Tim-1) to be the phenotypic marker for most IL-10^+^ B cells and stimulating this population by anti-Tim-1 antibodies prolongs both cardiac and islet allograft survival^11^. Although the role of B cells in regulating immune responses in many inflammatory conditions is now broadly accepted, the exact mechanism by which they work, and which molecules are essential for their function, is still being elucidated.

Gal-1, a small lectin, is part of a family of over 15 members sharing high affinity for β-galactosides^18,19^. Treatment with dimeric Gal-1 has been shown to increase IL-10 mRNA and protein levels in activated and non-activated CD4^+^ and CD8^+^ T cells^20^. Furthermore, activation of peripheral blood mononuclear cells (PBMCs) with anti-CD3 and Gal-1 induces significantly higher levels of IL-10 than stimulation with anti-CD3 alone^21^. In addition, direct binding of Gal-1 to CD45 on activated Th cells promotes IL-10 production^22^. It has also been documented that blocking Gal-1 binding significantly reduced the inhibitory functions of human and mouse regulatory T cells (Tregs)^23^. Moreover, several studies have documented the major role that Gal-1 plays in inducing T cells apoptosis^24–27^. For example, Toscano *et al*. showed that exposing activated T helper (Th) cells to Gal-1 *in vitro* could selectively induce the apoptosis of pro-inflammatory Th cells including Th1 and Th17, but not Th2 cells^27^. Zuniga *et al*. have reported that exogenous Gal-1 produced by BCR activated B cells can induce T cell apoptosis^28^. Although the role of Gal-1 in the control of the immune responses mediated by T cells is well characterized, less is known about its function in B cells. Gal-1 is expressed by plasma cells and by Blimp1-GFP^low^ plasma cells that are not fully differentiated^29^. However, whether Gal-1 affects the differentiation of B cells into regulatory cells or their function is not known.

In this study, Gal-1^−/−^ mice were used to investigate the contribution of Gal-1 in the generation and function of regulatory B cells.

## Results

### The lack of Gal-1 expression in B cells reduces IL-10 and Tim-1 expression upon anti-CD40 stimulation while TNF-α expression is increased

**Figure 1:**
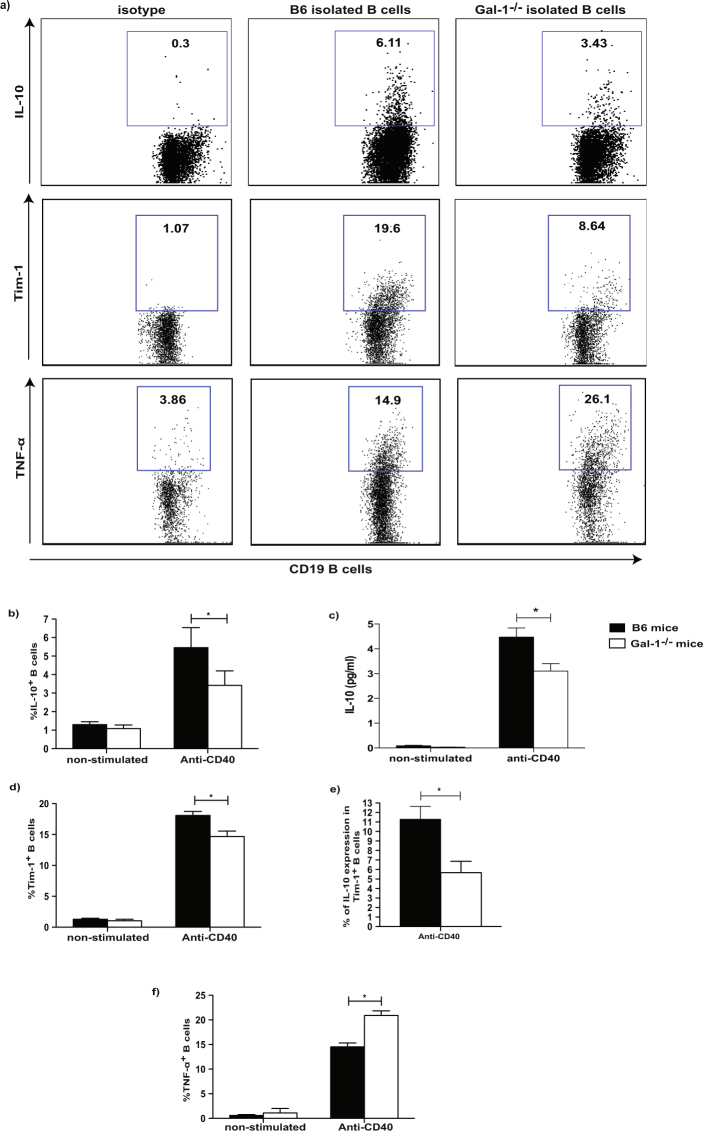
The lack of Gal-1 expression in B cells reduces IL-10 and Tim-1 expression upon anti-CD40 stimulation while TNF-α expression is increased. B cells were isolated from spleens of B6 & Gal-1^−/−^ mice by magnetic sorting and activated with anti-CD40 for 48 hrs. After collecting the supernatants, PMA, Ionomycin and brefeldin A were added for the last 4 hours of culture. B cells were then stained with anti-CD19, anti-IL-10, anti-Tim-1, and anti-TNF-α (ICC) Abs, and the supernatants were used to measure IL-10 production by CBA. (**a**) Representative FACS plots of IL-10, Tim-1 and TNF-α expression by anti-CD40 activated B cells that were isolated from WT B6 and Gal-1^−/−^ mice for 48 hrs. Histograms displaying, (**b**) IL-10 expression, (**c**) IL-10 production, (**d**) Tim-1 expression, (**e**) IL-10^+^ Tim-1^+^, (**f**) TNF-α expression on non-stimulated and stimulated B cells from WT B6 and Gal-1^−/−^ mice. Results represented as mean ± SEM, 4 independent experiments with 2 mice per group. Statistics were calculated by Mann-Whitney test, ^*^P < 0.05.

We have recently published that B cells from mice housed in the conventional animal facilities have regulatory function *in vitro* and *in vivo*^10^. To understand whether Gal-1 plays a role in the regulatory function of B cells, IL-10 and Tim-1 expression in B cells isolated from WT (C57BL/6 (B6)) mice and Gal-1^−/−^ mice housed under the aforementioned condition were analysed. It has been previously shown that CD40 stimulation is required for IL-10 production and Tim-1 expression by B cells^30–32^, both molecules have been identified as markers for regulatory B cells^11,33^. Splenic B cells were isolated from WT and Gal-1^−/−^ mice, stimulated *in vitro* with anti-CD40 antibody, and IL-10 expression and production as well as Tim-1 expression were assessed by flow cytometry. T and B cell purity is shown in Supplemental Fig. 1a. Gal-1^−/−^ B cells showed a reduction in IL-10 expression and production as well as Tim-1 expression compared to WT B cells (Fig. 1a–d). To further support the association between Tim-1 and IL-10, we assessed IL-10 expression by Tim-1^+^ B cells from either Gal-1^−/−^or WT mice and, as shown in Fig. 1e, IL-10 expression by Gal-1^−/−^ Tim-1^+^ B cells was also significantly reduced compared to WT Tim-1^+^ B cells.

TNF-α has been documented to promote the production of other pro-inflammatory cytokines by the immune cells, to promote tissue damage^34–37^, and has been reported to inhibit IL-10 induction^38^. We examined TNF-α expression in B cells purified from Gal-1^−/−^and WT mice and found that Gal-1^−/−^ B cells expressed significantly higher levels of TNF-α compared to WT B cells (Fig. 1a and f). Taken together these results suggest that Gal-1 deficiency in B cells shifts the balance between regulatory and pro-inflammatory cytokines towards an inflammatory response.

### Gal-1 expression by B cells is necessary for their acquisition of regulatory function to prolong allograft survival

**Figure 2:**
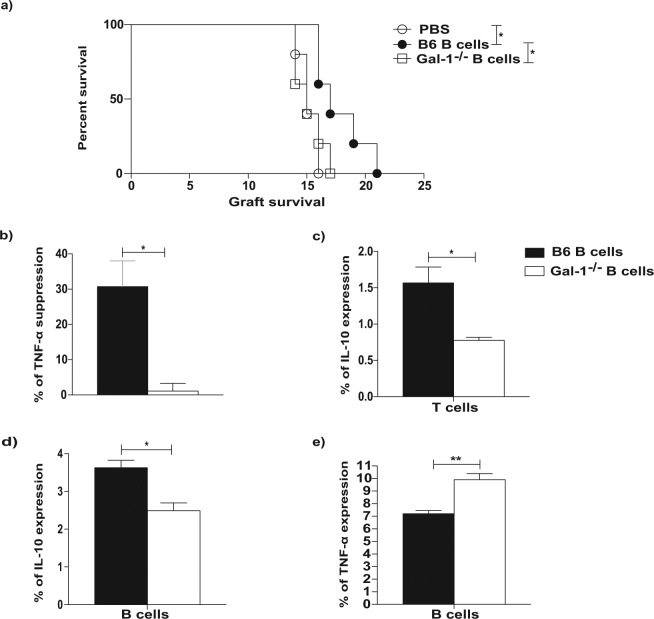
The Gal-1 expressions in B cells is necessary for their acquisition of regulatory function to prolong allograft survival. (**a**) CD8 depleted B6 (H2-K^b^) mice received dorsal skin grafts from B6 K^d^ (H2-K^d^) mice. One day prior to skin graft mice received 10 × 10^6^ B cells that were isolated from naïve B6, Gal-1^−/−^ mice, or PBS. Mice were monitored daily for rejection and grafts were said to be rejected when they formed hard black eschars. n ≥ 5/group for each group. Mean survival in days is denoted between parentheses. (**b**–**e**) B cells were isolated from spleens of B6 and Gal-1^−/−^ mice by magnetic sorting. B cells were co-cultured with negatively sorted CD4^+^ T cells isolated from B6 mice and irradiated allo-DCs (25 CD4 T cells: 1 allo-DCs) for 48 hrs. PMA, Ionomycin and brefeldin A were added for the last 4 hours of culture. Histograms displaying the percentage of, (**b**) suppression of TNF-α expressing CD4^+^ T cells, (**c**) IL-10 expressing CD4^+^ T cells, (**d**) IL-10 expressing B cells, (**e**) TNF-α expressing B cells. Results represented as mean ± SEM, 4 independent experiments with 10 mice per group. Statistics were calculated by log-rank (Mantel-Cox) test + Bonferonni correction for multiple comparisons and Mann-Whitney test, ^*^P < 0.05 & ^**^P < 0.005.

Having shown the importance of Gal-1 for IL-10 and TNF-α expression by B cells *in vitro*, we next evaluated the effect of Gal-1 on B cells *in vivo*. We have previously shown that the transfer of B cells obtained from conventionally housed WT mice can prolong MHC-class I mismatched skin allograft survival^10^. Thus, we tested whether Gal-1^−/−^ B cells could prolong allograft survival in the same skin graft model. Briefly, splenic B cells (1 × 10^7^ cells) isolated from conventionally housed naïve Gal-1^−/−^ and WT mice were adoptively transferred intravenously (i.v.) separately into naïve B6 mice one day before these recipient mice received skin grafts from transgenic B6 mice expressing H-2K^d^ (C57BL/6 mice express H-2K^b^)^39^. To avoid direct allorecognition by CD8^+^ T cells we used an anti-CD8 antibody, as previously described^39^. While B cells from WT mice prolonged skin graft survival, as we have previously published^10^, the transfer of B cells obtained from Gal-1^−/−^mice was unable to prolong skin transplant survival (Fig. 2a).

To further investigate the difference in the regulatory function between B cells isolated from WT and Gal-1^−/−^ mice, B cells were tested *in vitro* for their ability to inhibit CD4^+^  T cell allo-immune responses, as measured by TNF-α expression. B cells isolated from the spleens of Gal-1^−/−^ or WT mice were co-cultured with CD4^+^ T cells isolated from WT mice in the presence of irradiated allo-DCs for 48 hours. Only B cells isolated from WT but not Gal-1^−/−^ mice could suppress TNF-α expression by CD4^+^ T cells (Fig. 2b). Moreover, unlike WT B cells, Gal-1^−/−^ B cells were not able to induce IL-10 expression by CD4^+^ T cells (Fig. 2c). In addition, under the same culture conditions, we confirmed that B cells isolated from Gal-1^−/−^ mice expressed lower levels of IL-10 and higher levels of TNF-α compared to WT B cells (Fig. 2d and e). These results suggest that Gal-1 expression by B cells is required for the generation of IL-10 expressing regulatory B cells that can suppress allo-immune responses both *in vivo* and *in vitro*.

### The defect in the regulatory function of B cells from Gal-1^−/−^ mice is due to the defective function of T2 and T1 B cells

**Figure 3:**
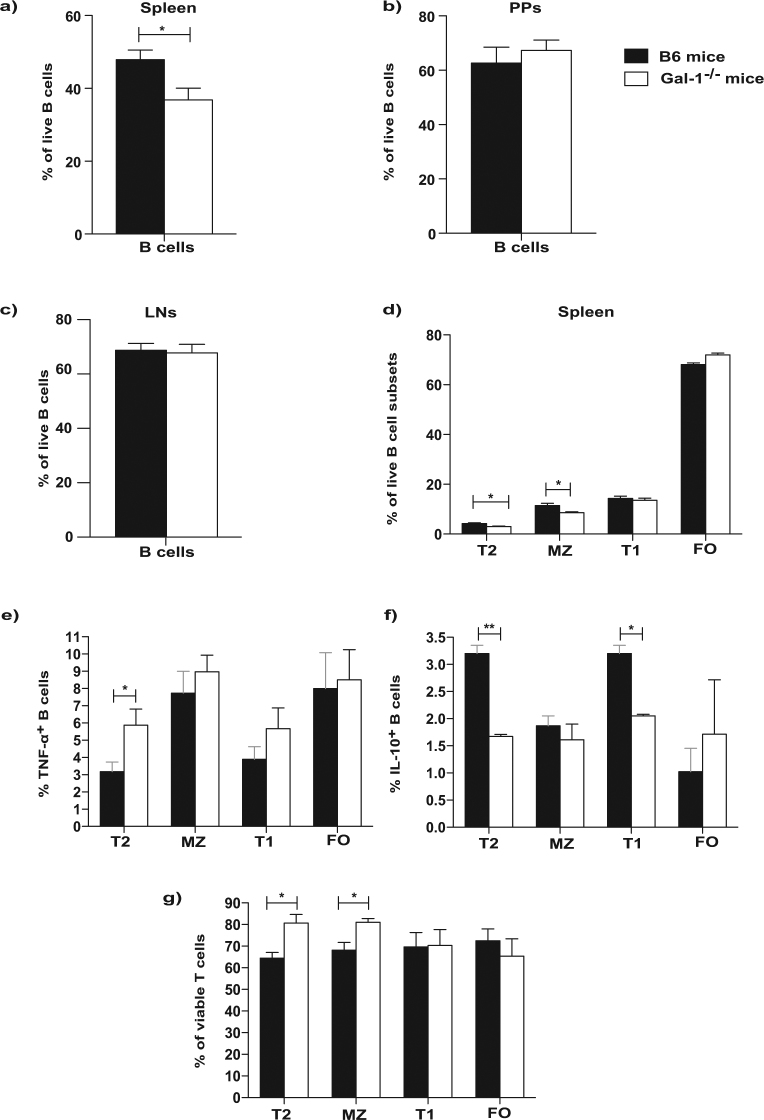
The defect in the regulatory function of B cells from Gal-1^−/−^ mice is due to the defective function in T2 and T1 subsets. (a–d) Spleens, PPs and LNs were isolated from naïve B6 and Gal-1^−/−^ mice, and live cells were phenotyped using the following Abs: anti-CD19, anti-CD21, anti-CD24, and anti CD23. Histograms displaying the percentages of, (**a**) total B cells in the spleens (n = 10), (**b**) total B cells in the PPs (n = 4), (**c**) total B cells in the LNs (n = 4), (**d**) B cell subsets in the spleens isolated from B6 and Gal-1^−/−^ mice (n = 4). Experiments were done independently with 1 mouse per group. (**e**,**f**) B cells were isolated from spleens of B6 and Gal-1^−/−^ mice maintained in CV facilities by magnetic sorting. B cell subsets were purified by FACS and co-cultured with negatively isolated WT CD4^+^ T cells and irradiated allo-DCs (25 CD4 T cells: 1 allo-DCs) for 48 hrs. PMA, Ionomycin and brefeldin A were added for the last 4 hrs of culture. Subsequently, cells were incubated with live/dead viability, anti-CD19, anti-CD4 mAbs (surface), and anti-TNF-α and anti-IL-10 mAbs (ICC). Histograms displaying the percentage of, 4 independent experiments with 10 mice per group (**e**) TNF-α expression by B cells, (**f**) IL-10 expression by B cells, (**g**) viable T cells. Results represented as mean ± SEM, statistics were calculated by Mann-Whitney test, ^*^P < 0.05 & ^**^P < 0.005.

Having shown that in the absence of Gal-1 B cells lost their regulatory capacity, next we analysed the composition of the B cells subsets in Gal-1^−/−^ compared to WT mice. Firstly, we observed a lower percentage of B cells in the spleens of Gal-1^−/−^ mice compared to WT mice (Fig. 3a), while no differences were observed in the percentages of B cells found in the Peyer patches (PPs) and Lymph node (LNs) (Fig. 3b and c). We next analysed the proportions of the different B cell subsets in the spleens, PPs and LNs (mandibular and axillary). The gating strategies for identifying the different B cell subsets are shown in the Supplemental Fig. 2a. Analysis of the different subsets of B cells revealed that the proportion of T2 and MZ B cells, both of which have been shown to be regulatory in other models, were decreased as percentages of total Gal-1^−/−^ B cells compared to WT B cells, whereas effector (FO) B cells were slightly increased in the spleens of Gal-1^−/−^ mice (Fig. 3d). Thus, the loss of total B cell suppression may be down to the loss of these subsets. LNs and PPs do not express T2 or MZ B cells; therefore, there were no differences in the percentages of B cell subsets between WT and Gal-1^−/−^ B cells isolated from them (data not shown).

To understand whether the differences in the function of B cells obtained from WT and Gal-1^−/−^ mice was simply due to the numerical impairment in the proportions of B cells in the spleens of Gal-1^−/−^ mice, particularly IL-10 producing regulatory B cells, B cell subsets were purified and tested both *in vitro* and *in vivo*. Splenic B cells were magnetically isolated from WT and Gal-1^−/−^ mice. Subsequently, B cell subsets were FACS sorted and added to co-culture of CD4^+^ T cells (isolated from naïve B6 mice) and allo-DCs for 48 hours. PMA, Ionomycin and brefeldin A were added for the last 4 hours of culture to analyse T cell survival, as well as TNF-α and IL-10 expression in B cell subsets. We found that T cell survival was enhanced when they were co-cultured with Gal-1^−/−^ T2 or MZ B cells compared to their counterpart WT B6 B cells. However, Gal-1^−/−^ T2 and T1 B cells, but not MZ B cells, co-cultured with CD4^+^ T cells stimulated with allo-DCs expressed higher levels of TNF-α and lower levels of IL-10 compared to WT T2 and T1 B cells (Fig. 3e,f and g).

**Figure 4:**
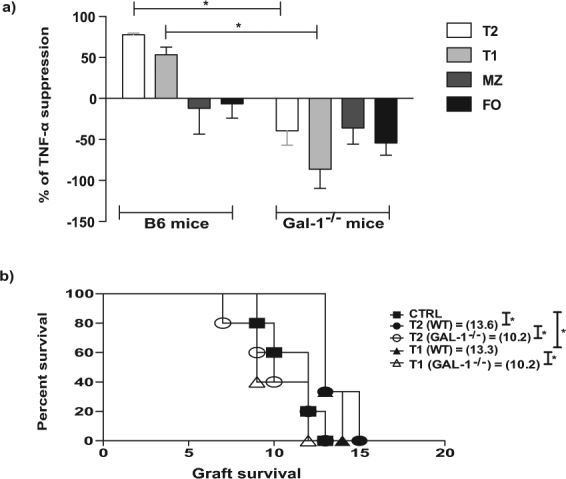
The defect in the regulatory function of B cells from Gal-1^−/−^ mice is due to the defective function inT2 and T1 subsets. (**a**) B cells were isolated from spleens of B6 and Gal-1^−/−^ mice maintained in CV facilities by magnetic sorting. B cell subsets were purified by FACS and co-cultured with negatively isolated WT CD4^+^ T cells and irradiated allo-DCs (25 CD4 T cells: 1 allo-DCs) for 48 hrs. PMA, Ionomycin and brefeldin A were added for the last 4 hrs of culture. Subsequently, cells were incubated with anti-CD19 and anti-CD4 mAbs (surface), and anti-TNF-α mAbs (ICC). Histogram displaying the percentage of suppression of TNF-α expressing WT CD4^+^ T cells, of 4 independent experiments with 10 mice per group. (**b**) CD8 depleted B6 (H2-K^b^) mice received dorsal skin grafts from B6 K^d^ (H2-K^d^) mice. One day prior to skin graft mice received 1 × 10^6^ B cell subsets that were isolated from B6 mice (n = 3/group for each experiment), Gal-1^−/−^ mice (n = 5/group for each experiment), or PBS (n = 5/group for each experiment). Mice were monitored daily for rejection and grafts were said to be rejected when they formed hard black eschars. Mean survival in days is denoted between parentheses. Results represented as mean ± SEM, Statistics were calculated by log-rank (Mantel-Cox) test + Bonferonni correction for multiple comparisons and Mann-Whitney test, ^*^P < 0.05.

We further analysed the suppressive function of the B cell subsets isolated from Gal-1^−/−^ mice by testing their suppressive ability *in vitro* (Fig. 4a). Gal-1^−/−^ T2 and T1 B cells were unable to suppress TNF-α expression by CD4^+^ T cells compared to WT counterparts (Fig. 4a). In agreement with our previous publication^10^, MZ B cells failed to suppress even when isolated from WT mice, however, in accordance with their decrease in IL-10 expression (Fig. 3f), the lack of Gal-1 expression appeared to cause the MZ B cells to enhance CD4^+^ T cell TNF-α expression (Fig. 4a). We confirmed that Gal-1^−/−^ T2 and T1 B cells had lost their regulatory capacity *in vivo* by testing their ability to inhibit MHC class-I mismatched skin allograft survival following adoptive transfer to B6 recipients. Neither Gal-1^−/−^ T1 nor T2 B cells were able to prolong skin allograft survival, while their WT counterparts were able to do so (Fig. 4b). These results indicate that Gal-1 expression is required for regulatory B cell function, particularly for T2 and T1 regulatory B cells.

## Discussion

Mounting data suggests the existence of B cell populations with regulatory function such as T2 and T1 B cells^11–16^. These cells have been reported to suppress several autoimmune diseases and to prolong allograft survival^10,12,17^. In this study we show that Gal-1 plays an important role in the induction of regulatory function by B cells both *in vitro* and *in vivo*. In an *in vivo* model of skin transplant, unlike B cells derived from WT mice, the adoptive transfer of B cells from Gal-1^−/−^ mice did not prolong the survival of MHC-class I mismatched skin allograft. Furthermore, Gal-1^−/−^B cells, unlike WT B cells, did not suppress TNF-α expression by T cells stimulated by allo-DCs *in vitro*. Furthermore, Gal-1 expression on CD40-activated B cells appears to be required for IL-10 and Tim-1 expression, as Gal-1^−/−^ B cells have reduced expression of IL-10 and Tim-1 as well as IL-10 production. The loss of Gal-1 on B cells was also associated with an increased expression of TNF-α compared to WT B cells suggesting that Gal-1 molecule may play a role in ‘polarisation’ of the B cells cytokine profiles.

Recently a role for Gal-1 has been demonstrated in B cell development as well. The first checkpoint in B cells differentiation requires the clustering of their pre-BCR a process induced by Gal-1 binding^40^. In our study, using Gal-1^−/−^ mice allowed us to study the influence of Gal-1 on the development of B cells both numerically and functionally. We have observed in this study a lower percentage of B cells, particularly the regulatory T2 B cells in the spleens of Gal-1^−/−^ mice compared to WT mice (Fig. 3a), while no differences were observed in the percentages of B cells found in the PPs and LNs. It has been reported that bone marrow (BM) B cells do not express Gal-1, however, Gal-1 produced by BM stromal cells play an important role in B cell differentiation and proliferation^41^. Espeli and colleagues showed that co-culturing stromal cells with pro-B/pre-B cells and inhibiting the interaction of pre-BCR with Gal-1 significantly impaired B cell proliferation, and decreased the numbers of pre-B and immature B cells^41^. These observations might explain the decrease we found in the percentage of B cells in the spleens of Gal-1^−/−^ mice. Nevertheless, the differences seen in the spleen were not the same of what was published by Clark *et al*. They showed that the number of B220^+^ cells in the spleen of Gal^−/−^ mice were equivalent to WT animals which might be due to the housing conditions, as the knockout mice were housed in SPF facilities, while the mice used in our study were housed in the CV facilities of the animal house. Importantly and different from us they did not assess the different B cell subsets^42^.

Our data suggests that Gal-1 expression plays a role in the induction of transitional B cell subsets regulative capacity. In fact, Gal-1^−/−^ T2 and T1 B cells were not able to suppress CD4^+^ T cells *in vitro* and to prolong skin allograft survival. Several physiological mechanisms of immune regulation have been known to suppress effector T cells such as IL-10 and induction of apoptosis^43,44^. Various studies have reported the role of Gal-1 in the induction of IL-10^20-22^; for example the treatment of activated T cells with dimeric Gal-1 increased IL-10 mRNA levels^20^. Although most of these studies have used recombinant Gal-1 protein, here we show that Gal-1 deficiency influence the expression of IL-10 and TNF-α by B cells during their development. Our data show that in particular the loss of Gal-1 affected the cytokine profile of Transitional B cell subsets. In our previous publication^10^, we have shown that T2 and T1 B cell subsets were regulatory only when isolated from mice housed in environment that has no special precautions to prevent entering of infectious agents and that their regulatory function was dependent on IL-10. In this study using the same experimental conditions, we found that IL-10 expression in the Gal-1^−/−^ Transitional B cells was significantly lower compared to the same two subsets derived from WT mice, the same for TNF-α expression although mostly in Gal-1^−/−^ T2 and only slightly in T1 B cells. In contrast, the cytokine expression of the MZ B cells was not affected. Whether the effect observed was due to differences in the amount of Gal-1 expressed or produced by these B cell subsets or by other cells and the localisation of the Gal-1 in the different cell types are unknown and need further investigation.

A previous study reported that exogenous Gal-1 produced by BCR activated B cells can induce T cell apoptosis^28^. In our co-culture experiments, increased T cell survival occurred in the presence of Gal^−/−^ B cells. Although we did not measure T cell apoptosis directly, nor have we shown that anti-CD40 activated B cells produce Gal-1 it is tempting to suggest that the observation by Zuniga et al. is happening in our cultures^28^. However, our observations are purely circumstantial and need to be validated. What is also interesting but we do not have a mechanism for this observation is that survival of T cells is influenced by the Gal^−/−^ B cell subsets T2 and MZ but not by T1 B cells. This result again suggests that Gal-1 not only influence the functional development of B cells, but that it does so in a different manner between the different B cell subsets.

Collectively, we have provided some evidence that Gal-1 affects the function of B cells, in particular Transitional Bregs, affecting their cytokine expression and their suppressive capacity. However, whether Gal-1 plays its role extracellularly or intracellularly needs further investigation.

## Materials and Methods

### Ethics Statement

These studies were approved and conducted in accredited facilities in accordance with The Home Office UK Animals (Scientific Procedures) Act 1986 (Home Office license number PPL 70/7302).

### Mice

Wild type C57BL/6 (H-2K^b^) and BALB/c (H-2d) mice were purchased from Harlan Laboratories. B6K^d^ mice were a generous gift from Dr. R Pat Bucy (University of Alabama at Birmingham, Birmingham, AL, USA). Gal-1^−/−^ mice (on C57BL/6 background) were kindly provided by F. Poirier. All mice groups were maintained in conventional (CV) facilities for the same period of time (4 weeks). The Home Office (UK) approved all mouse protocols utilized in this study.

### Purification of B cells, B cell subsets and CD4^+ ^T cells

Splenic B cells were purified by negative selection using CD43 microbeads (Miltenyi Biotech, 130–049801) according to manufacturer’s instructions. Splenic CD4^+^ T cells were isolated using Invitrogen Dynabeads® Untouched™MouseCD4 Cells kits, according to manufacturer’s instructions (Invitrogen, 11416D). Purified splenic B cells were stained with anti-CD21, anti-CD24 and anti-CD23 antibodies. Cells were then incubated for 30 mins at 4 °C, and washed 2 times. Next, cells were re-suspended in 1 ml of FACS buffer. DAPI was added to the cells just before starting the sort to exclude dead cells. Sorting took place on a BD FACSAria (BD Biosciences).

### B cell activation system

Magnetically isolated B cells (2 × 10^5^ cells/well) were activated for 48 hours with anti-CD40 antibodies (10 µg/ml) from FGK45 Hyridoma. After collecting the supernatants, PMA, Ionomycin and brefeldin A were added for the last 4 hours of culture. Next, the cells were labelled with surface antibodies and stained for intracellular cytokines, and the supernatants were used to measure IL-10 production by CBA.

### DCs generation

DCs were generated from bone marrow as previously described^39^. One day before DC isolation, 100ng/mL LPS (E.coli, EnzoLife Sciences, UK) was added to induce maturation. At day 7 of culture, DCs were collected, washed three times, and irradiated.

### *In vitro* suppression assays

Isolated CD4^+^ T cells were co-cultured with irradiated allo-DCs alone at a ratio of 25 T cells: 1 DC, or with B cells and allo-DCs at a ratio of 25 T cells: 25 B cells: 1DC. Cells were cultured for 48 hrs at 37 °C in RPMI in 96 well plates (1.5–2 × 10^5^ cells/well).

### Skin transplants

Donor tail skin grafts were performed and monitored as previously described^39^. Anti-CD8 antibody (clone YTS169, 250 μg/injection/mouse) was injected i.p. at day-1 and day 1 after skin graft, and weekly thereafter. B cells (1 × 10^7^cells) and B cell subsets (1 × 10^6^cells) were injected intravenously one day after transplant.

### Antibodies and Flow Cytometry

All FACS antibodies were purchased from eBioscience. Surface staining was performed as previously described^39^. For intracellular staining, cells were stained then fixed/permebilized according to the eBioscience protocol. Permeabilized cells were incubated in Permwash^TM^ with anti-IL10, anti-TNF-α or appropriate isotype control for 30 mins at 4 °C (with blocking anti-CD16/32). For the detection of intracellular cytokines, PMA (50ng/ml), ionomycin (1 μM) and brefeldin A were added for 5 hrs before staining. Cells were acquired using LSRII or Fortessa flow cytometers (BD Biosciences). FlowJo software was used for analysis.

### Cytomeric Beads Arrays

The levels of IL-10 production were measured by CBA assay (CBA, BD, Oxford, UK) according to the manufacturer’s instructions. Briefly, IL-10 capture beads were added to the detection reagent and to 50 μl from the supernatants, which were collected after stimulating B cells isolated from Gal-1^−/−^ and B6 mice with anti-CD40 Abs (non-stimulated B cells were used as controls) for 48 hrs. After 2 hrs of incubation, the beads were washed and resuspended. Beads were acquired using Fortessa flow cytometer (BD Biosciences). FCAP Array software was used for analysis.

### Statistical analysis

Comparisons between groups were performed using Mann-Whitney test for two groups. Log Rank (Mantel-Cox) tests were used for graft survival curves. Analyses were performed using GraphPad Prism software.

## Electronic supplementary material

Supplementary Figure 1, Figure 2
