# Supplementary material for: Galectin-1 is required for the regulatory function of B cells
Source: Sci Rep. 2018 Feb 9;8:2725. doi: 10.1038/s41598-018-19965-z (PMC5807431; doi:10.1038/s41598-018-19965-z)
Supplement: Supplementary file 1 — Supplementary Figure 1, Figure 2 [file 41598_2018_19965_MOESM1_ESM.doc]

**Galectin-1 is required for the regulatory function of B cells**

R. Alhabbab^1,2§^, P. Blair^2,3§^, L. Smyth^2,4§^, K. Ratnasothy^2§^, Q. Peng^2^, A. Moreau^2,5^, R. Lechler^2^, R. Elgueta^2, §^and G.  Lombardi^2, §*^

Al Habbab et al. Supplemental Figure 1


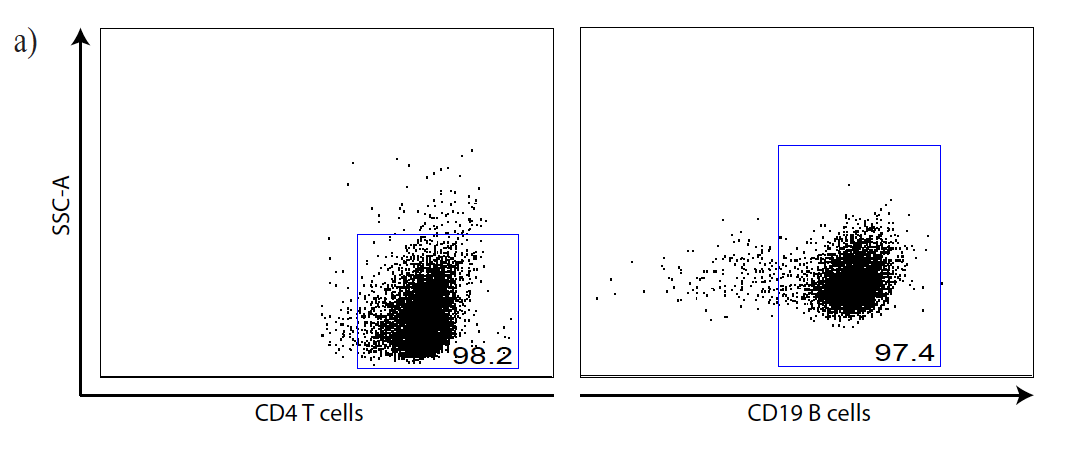


**Supplemental Figure 1 The lack of Gal-1 expression in B cells reduces IL-10 and Tim-1 expression upon anti-CD40 stimulation while TNF-αproduction is increased.**T and B cells were isolated from spleens of the mice by magnetic sorting. T cells were then stained with anti-CD4 and B cells were stained with anti-CD19Abs to check their isolation purity. (a) Representative FACS plots of T cells (on the left), and B cells (on the right).

Al Habbab et al. Supplemental Figure 2


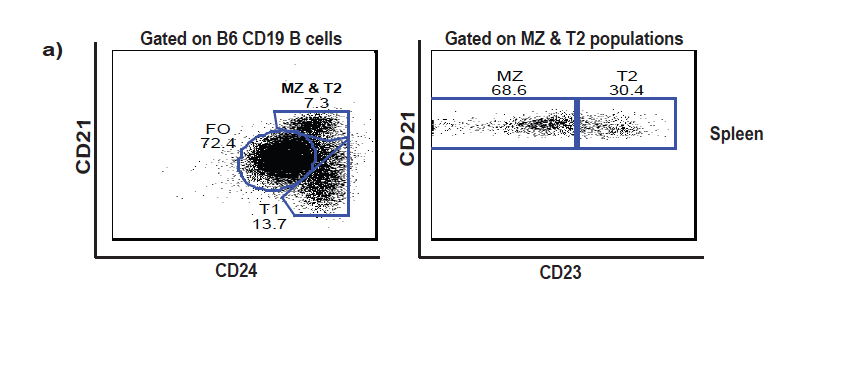


**Supplemental Figure 2 The defect in the regulatory function of B cells from Gal-1^-/-^ mice is due to the defective function in T2 and T1 subsets.** Spleens, PPs and LNs were isolated from naïve B6 and Gal-1^-/-^ mice, and phenotyped using the following Abs: anti-CD19, anti-CD21, anti-CD24, and anti-CD23. (a) Representative FACS plots of B cell subsets gating strategies in the spleens.
